# A retrospective analysis of cisplatin, pemetrexed, and bevacizumab in previously treated non-small-cell lung cancer

**DOI:** 10.18632/oncotarget.4262

**Published:** 2015-06-11

**Authors:** Ke-Jun Liu, Hai-Ying Wu

**Affiliations:** ^1^ State Key Laboratory of Oncology in South China, Collaborative Innovation Center of Cancer Medicine, Sun Yat-Sen University Cancer Center, Guangzhou, China; ^2^ Department of Medical Oncology, Dongguan People's Hospital, Dongguan, China

**Keywords:** non-small-cell lung cancer (NSCLC), bevacizumab, platinum-based chemotherapy

## Abstract

Patients with non-small-cell lung cancer (NSCLC) often have an advanced disease when firstly diagnosed. Bevacizumab is a monoclonal antibody against vascular endothelial growth factor receptor (VEGFR). In this study, we retrospectively analyzed the efficacy of cisplatin, pemetrexed, and bevacizumab in previously treated advanced NSCLC. Results showed that the objective response rate(ORR) of this novel regimen is 43%, median progression-free survival (PFS) was 5.2 months (95% CI, 3.7 to 6.7 months) and median overall survival (OS) was 11.4 months (95% CI, 8.8 to 13.9 months). Adverse events were generally mild, ranging from grade 1 to grade 3. In conclusion, the combination of cisplatin, pemetrexed, and bevacizumab obtained promising results in selected patients with NSCLC. Randomized clinical trials are needed to further investigate the efficacy of this regimen.

## INTRODUCTION

Non-small-cell lung cancer (NSCLC) is one of the most common carcinoma worldwide [1]. The prognosis of patients with advanced NSCLC is poor, with a median progression-free survival (PFS) of 4–6 months and median overall survival (OS) of 8–10 months [2–7]. Patients harbor epidermal growth factor receptor (EGFR) mutations may benefit from treatment with tyrosine kinase inhibitors (TKIs) such as erlotinib [8] and gefitinib [9]. However, for patients with EGFR wild-type NSCLC, platinum-based chemotherapy is still used for front line treatment.

Angiogenesis is necessary for cancer cells to proliferate and metastasize. The vascular endothelial growth factor (VEGF) could promote tumor angiogenesis [10–12]. Previous studies indicate that VEGF is over expressed in several malignant tumors [13, 14]. Bevacizumab is a monoclonal antibody against VEGF receptor, hence exerts antitumor effect by inhibiting abnormal vascular growth in malignant tumors [15–18]. When adding bevacizumab to platinum-based chemotherapy in the E4599 study, the median OS of nonsquamous NSCLC patients was prolonged to one year (12.3 months), with relatively tolerable toxicities [19]. Based on results from the above study, the U.S. Food and Drug Administration (FDA) approved the use of bevacizumab as first-line therapy for advanced NSCLC [20].

Till date, only single antitumor agents such as erlotinib [21], docetaxel [22, 23] and pemetrexed [24], are recommended in second line therapy. Platinum-based chemotherapy combined with bevacizumab may be effective if patients failed in previously first-line therapy of erlotinib or crizotinib. However, several studies investigated the efficacy of bevacizumab combined with chemotherapeutical drugs for previously treated NSCLC [25–29]. These studies showed increased objective response rate (ORR) and enhanced PFS when compared with the standard second-line therapy.

The regimen of cisplatin plus pemetrexed is extensively used for advanced nonsquamous NSCLC in the clinic [30–36]. This combination produced superior effect than that of cisplatin plus gemcitabine. In addition, results from a phase III study showed improved efficacy when adding bevacizumab to this regimen in front line treatment [37]. Currently, there are no reports concerning the combination of cisplatin, pemetrexed, and bevacizumab for advanced NSCLC beyond first-line settings. Hence, we retrospectively analyzed this regimen for NSCLC patients in our Cancer Center.

## RESULTS

### Patient characteristics and treatment

The number of eligible patients in our study was 7. The clinical characteristics of these patients are listed in Table 1. Among all the patients, 5 patients (71%) were < 60 years of age (median 50 years; range 28–63 years) and 6 patients (86%) were male. Most patients had a performance status of 1 score and adenocarcinoma subtype (both 86%). Two patients (29%) were EGFR gene mutated, three patients (42%) were wild-type and the remaining two patients (29%) were not taken gene sequencing. Patients had received at least one line of therapy before the initial treatment. In our study, all patients received bevacizumab (7.5 mg/kg), cisplatin (75 mg/m^2^) and pemetrexed (500 mg/m^2^) administered every 3 weeks. Dexamethasone, folic acid and vitamin B12 were administered routinely and treatment continued until patients having a progressed disease. Treatment sustained for at least 2 cycles or until disease progression or unacceptable toxicity or economic factors. A median of 4 cycles were administered in this study.

**Table 1:** Patient characteristics

Patients Characteristic	*n*	(%)
Age		
Median	50	
Range	28–63	
Years		
18–60	5	(71%)
60–70	2	(29%)
Sex		
Male	6	(86%)
Female	1	(14%)
ECOG PS		
0	1	(14%)
1	6	(86%)
Smoking history		
Yes	4	(57%)
No	3	(43%)
Pathological type		
Adenocarcinoma	6	(86%)
Large cell	1	(14%)
No. prior regimens		
1	1	(14%)
2	3	(43%)
3	2	(29%)
4	1	(14%)
EGFR status		
Mutation	2	(29%)
Wild-type	3	(42%)
NOS	2	(29%)

### Efficacy

**Figure 1:**
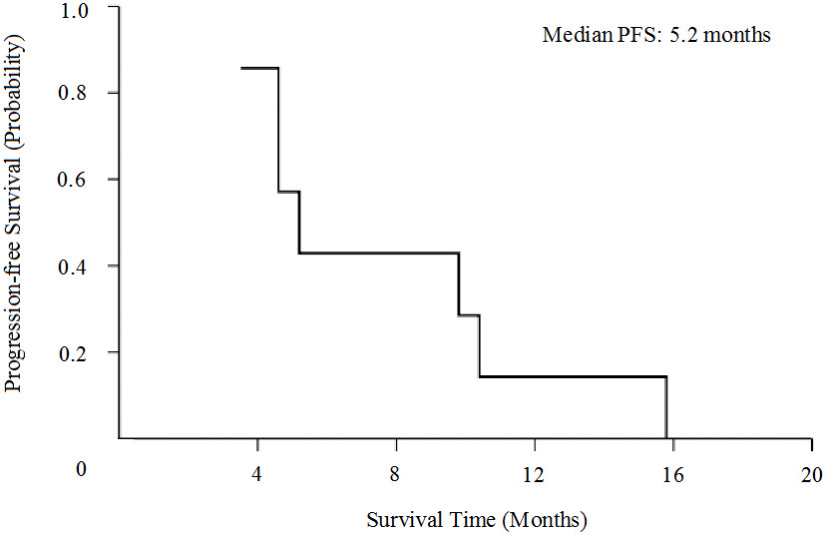
Kaplan-Meier curves for progression-free survival (PFS)

**Figure 2:**
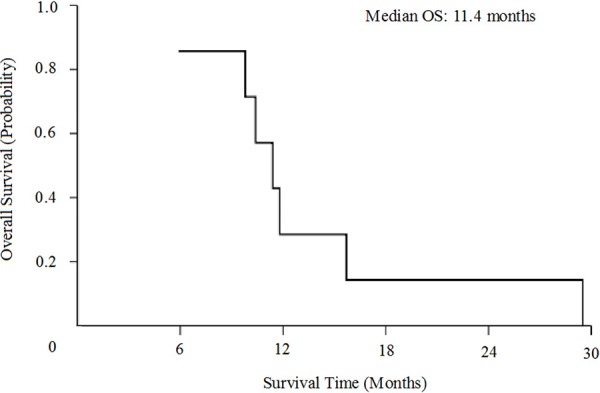
Kaplan-Meier curves for overall survival (OS)

As is illustrated in Table 2, disease control of this novel combination was observed in 6 of 7 NSCLC patients in the study (43% for PR and 43% for SD). Objective response rate was 43%. Only one patient obtained PD after 2 cycle of therapy. Median PFS was 5.2 months (95% CI, 3.7 to 6.7 months, Figure 1) and median OS was 11.4 months (95% CI, 8.8 to 13.9 months, Figure 2).

**Table 2:** Efficacy results

Variable	No	%
Response		
PR (%)	3	43
SD (%)	3	43
PD (%)	1	14
Median PFS (months)	5.2	
95% CI	3.7 to 6.7	
Median survival (months)	11.4	
95% CI	8.8 to 13.9	

### Adverse events

Main toxicities possibly related to therapy are listed in Table 3. Adverse events of this chemotherapeutical regimen were generally mild, ranging from grade 1 to grade 3. Hematologic toxicities observed in the study were mainly grade 1. The most common grade 2 adverse events were non-hematologic toxicities, including 4 episodes of nausea and 3 episodes of anorexia and fatigue. Grade 3 toxicities were anorexia (42.9%), fatigue (28.6%) and nausea (14.3%). No patients in the study had severe adverse events.

**Table 3:** Treatment-related adverse events

Toxicity	Grade 1	Grade 2	Grade 3
Neutropenia	1	0	0
Thrombocytopenia	1	0	0
Anemia	4	1	0
Dizziness	3	1	0
Fever	1	0	0
Infection	1	2	0
Bleeding	3	0	0
Fatigue	0	3	2
Nausea	0	4	1
Vomiting	0	2	0
Alopecia	1	1	0
Anorexia	1	3	3
Dyspnea	3	1	0
Constipation	1	2	0
Cough aggravation	3	1	0
Abdominal pain	1	1	0
Diarrhea	1	0	0
Rash	1	0	0
Pruritus	0	1	0
ALT ↑	2	0	0
Thirst	4	1	0
Insomnia	0	0	0

## DISCUSSION

Standard second-line treatments for patients with advanced NSCLC are mainly single agents. For third-line or beyond therapy, possible choices are erlotinib (regardless of EGFR gene status), crizotinib (if ALK fusing gene existed), clinical trial or best support therapy. Bevacizumab showed encouraging efficacy as first-line therapy for nonsquamous NSCLC patients. In the AVAPEAL study, the treatment results were increased when adding bevacizumab to the combination of cisplatin plus pemetrexed.

Bevacizumab showed increased efficacy in previously treated advanced NSCLC when combined with erlotinib [38, 39]. In one study, the response rates of bevacizumab plus erlotinib was 51.3%, median PFS and OS was 4.4 and 13.7 months, respectively [39]. The combination of bevacizumab, oxaliplatin and pemetrexed for previously treated NSCLC was also investigated [26]. Clinical beneficial rate was 71% and median PFS and OS was 5.8 and 12.5 months, respectively. These studies suggested that bevacizumab may benefit patients with advanced nonsquamous NSCLC in second-line or beyond settings.

In our former study, we investigated the chemotherapeutic regimen of pemetrexed plus bevacizumab in previously treated NSCLC [29]. Results showed that disease control rate was 54.84%, median PFS was 4.37 months and median OS was 15.83 months. Toxicities of this combination treatment are generally tolerable. According to the results of above studies, we conducted this study analyzing the effect of cisplatin, pemetrexed, and bevacizumab for advanced nonsquamous NSCLC beyond first-line settings. This study showed encouraging findings, with ORR 43%, median PFS 5.2 months and median OS 11.4months. Toxicities were also manageable, which rarely produced grade 3 or higher adverse events. These results may possibly due to the young population and good performance status of patients in our study (all are ≤ 65 years and PS ≤ 1). As the number of patients in this study was relatively small, additional studies are needed to further evaluate the efficacy of this combination beyond first-line setting.

In the study, all patients received EGFR TKIs treatment before the combination therapy. The period ranged from 1 to 14 months, the median therapeutic time was 7 months. After failed from oral EGFR TKIs treatment, patients received salvage therapy of cisplatin, pemetrexed, and bevacizumab for a median time of 4 cycles. The median number of therapeutic time is third-line. Four patients took another EGFR TKIs after failed from this treatment, a strategy according to previous reports. One of the four patients was later found to be ALK fusing gene mutated and received crizotinib for about 1 year. Therefore, the combination regimen of cisplatin, pemetrexed, and bevacizumab enhanced overall survival of patients with advanced NSCLC.

In previous studies, the dose of bevacizumab as second-line or beyond was 15 mg/kg, such as that administered in the E4599 study. The AVAiL study compared the effect of bevacizumab in different doses (7.5 and 15 mg/kg) in combination with chemotherapy [40]. Results showed that both low and high dose level of bevacizumab enhanced median PFS compared to chemotherapy alone arm. In the later AVAPEAL study, researchers used the low dose level (7.5 mg/kg) and also produced favorable results [37]. Nowadays, there are no reports concerning 7.5 mg/kg bevacizumab plus chemotherapy for previously treated advanced NSCLC. In addition, the prescription of bevacizumab seems expensive for patients living in developing countries. Our study showed that the dose of 7.5 mg/kg bevacizumab also works when combined to cisplatin and pemetrexed in the second-line and beyond settings.

In conclusion, the combination of cisplatin, pemetrexed, and bevacizumab obtained promising efficacy in selected patients with previously treated NSCLC. More clinical trials are needed to further elaborate the relationship between this novel regimen and advanced NSCLC in sencon-line or beyond setting.

## MATERIALS AND METHODS

### Patients

We conducted a retrospective analysis of patients with previously treated nonsquamous NSCLC at Sun Yat-Sen University Cancer Center from December 2011 to September 2012. The eligible patients were ≥ 18 years old, with cytological or histological confirmation of stage IIIB (with pleural effusion) and stage IV nonsquamous NSCLC (The International Association for the Study of Lung Cancer 7^th^ edition of Tumor Node Metastasis Staging classification) and had already failed at least one platinum-based chemotherapy regimen. Patients whose clinical information could not be completely obtained were excluded from our analysis.

### Data collection

The clinical data of patients in our studies were collected carefully. All the patients had an ECOG PS of 0 to 1. Patient history, physical examination and complete blood work were recorded at baseline and before each cycle of treatment. Tumor response was evaluated by computed tomography scans according to Response Evaluation Criteria in Solid Tumors (RECIST) criteria. Disease control was defined as complete remission (CR), partial remission (PR) or stable disease (SD). Patients who had a progression disease after two cycles of treatment were defined as progression disease (PD). PFS was defined as time between the start of the treatment and disease progression or death. OS was defined as time between the start of the treatment and last contact or death. Toxicities were recorded and classified in the light of the National Cancer Institute Common Terminology Criteria for Adverse Events (NCI-CTCAE) version 3.0.

### Statistical analysis

Statistical analysis was performed by Statistical Product and Service Solutions (SPSS) 18.0 software. Estimates of PFS and OS were calculated using the Kaplan-Meier method and two-sided 95% confidence interval were obtained.

## Abbreviations

NSCLC: non-small-cell lung cancer
PFS: progression-free survival
OS: overall survival
EGFR: epidermal growth factor receptor
TKIs: tyrosine kinase inhibitors
VEGF: Vascular epidermal growth factor
FDA: the U.S. Food and Drug Administration
ORR: objective response rate
RECIST: Response Evaluation Criteria in Solid Tumors
CR: complete remission
PR: partial remission
SD: stable disease
PD: progression disease
NCI-CTCAE: National Cancer Institute Common Terminology Criteria for Adverse Events
SPSS: Statistical Product and Service Solutions

## References

[1] Jemal A, Bray F, Center MM, Ferlay J, Ward E, Forman D (2011). Global cancer statistics. CA Cancer J Clin.

[2] Schiller JH, Harrington D, Belani CP, Langer C, Sandler A, Krook J, Zhu J, Johnson DH (2002). Comparison of four chemotherapy regimens for advanced non-small-cell lung cancer. N Engl J Med.

[3] Scagliotti GV, De Marinis F, Rinaldi M, Crinò L, Gridelli C, Ricci S, Matano E, Boni C, Marangolo M, Failla G, Altavilla G, Adamo V, Ceribelli A (2002). Phase III randomized trial comparing three platinum-based doublets in advanced non-small-cell lung cancer. J Clin Oncol.

[4] Hotta K, Matsuo K, Ueoka H, Kiura K, Tabata M, Tanimoto M (2004). Addition of platinum compounds to a new agent in patients with advanced non-small-cell lung cancer. a literature based meta-analysis of randomised trials. Ann Oncol.

[5] Stinchcombe TE, Socinski MA (2009). Current treatments for advanced stage non-small cell lung cancer. Proc Am Thorac Soc.

[6] Zatloukal P, Petruzelka L, Zemanová M, Kolek V, Skricková J, Pesek M, Fojtů H, Grygárková I, Sixtová D, Roubec J, Horenková E, Havel L, Průsa P (2003). Gemcitabine plus cisplatin vs. gemcitabine plus carboplatin in stage IIIb and IV non-small cell lung cancer: a phase III randomized trial. Lung cancer.

[7] Liu KJ, Guan ZZ, Liang Y, Yang XQ, Peng J, Huang H, Shao QX, Wang MZ, Zhu YZ, Wu CP, Wang SB, Xiong JP, Bai YX (2014). A double-blind, randomized phase II study of dicycloplatin plus paclitaxel versus carboplatin plus paclitaxel as first-line therapy for patients with advanced non-small-cell lung cancers. Arch Med Sci.

[8] Perez-Soler R, Chachoua A, Hammond LA, Rowinsky EK, Huberman M, Karp D, Rigas J, Clark GM, Santabarbara P, Bonomi P (2004). Determinants of tumor response and survival with erlotinib in patients with non-small-cell lung cancer. J Clin Oncol.

[9] Kris MG, Natale RB, Herbst RS, Lynch TJ, Prager D, Belani CP, Schiller JH, Kelly K, Spiridonidis H, Sandler A, Albain KS, Cella D, Wolf MK (2003). Efficacy of gefitinib, an inhibitor of the epidermal growth factor receptor tyrosine kinase, in symptomatic patients with non-small cell lung cancer: a randomized trial. JAMA.

[10] Ferrara N (2002). Role of vascular endothelial growth factor in physiologic and pathologic angiogenesis: therapeutic implications. Semin Oncol.

[11] Folkman J, Merler E, Abernathy C, Williams G (1971). Isolation of a tumor factor responsible for angiogenesis. J Exp Med.

[12] Bergers G, Benjamin LE (2003). Tumorigenesis and the angiogenic switch. Nat Rev Cancer.

[13] Ferrara N, Gerber HP, LeCouter J (2003). The biology of VEGF and its receptors. Nat Med.

[14] Bremnes RM, Camps C, Sirera R (2006). Angiogenesis in non-small cell lung cancer: the prognostic impact of neoangiogenesis and the cytokines VEGF and bFGF in tumours and blood. Lung cancer.

[15] Hainsworth JD, Sosman JA, Spigel DR, Edwards DL, Baughman C, Greco A (2005). Treatment of metastatic renal cell carcinoma with a combination of bevacizumab and erlotinib. J Clin Oncol.

[16] Jenab-Wolcott J, Giantonio BJ (2009). Bevacizumab. current indications and future development for management of solid tumors. Expert Opin Biol Ther.

[17] Hurwitz H, Fehrenbacher L, Novotny W, Cartwright T, Hainsworth J, Heim W, Berlin J, Baron A, Griffing S, Holmgren E, Ferrara N, Fyfe G, Rogers B (2004). Bevacizumab plus irinotecan, fluorouracil, and leucovorin for metastatic colorectal cancer. N Engl J Med.

[18] Deng T, Zhang L, Liu XJ, Xu JM, Bai YX, Wang Y, Han Y, Li YH, Ba Y (2013). Bevacizumab plus irinotecan, 5-fluorouracil, and leucovorin (FOLFIRI) as the second-line therapy for patients with metastatic colorectal cancer, a multicenter study. Med Oncol.

[19] Sandler A, Gray R, Perry MC, Brahmer J, Schiller JH, Dowlati A, Lilenbaum R, Johnson DH (2006). Paclitaxel-carboplatin alone or with bevacizumab for non-small-cell lung cancer. N Engl J Med.

[20] Cohen MH, Gootenberg J, Keegan P, Pazdur R (2007). FDA drug approval summary: bevacizumab (Avastin) plus Carboplatin and Paclitaxel as first-line treatment of advanced/metastatic recurrent nonsquamous non-small cell lung cancer. Oncologist.

[21] Shepherd FA, Rodrigues Pereira J, Ciuleanu T, Tan EH, Hirsh V, Thongprasert S, Campos D, Maoleekoonpiroj S, Smylie M, Martins R, van Kooten M, Dediu M, Findlay B (2005). Erlotinib in previously treated non-small-cell lung cancer. N Engl J Med.

[22] Fossella F, Pereira JR, von Pawel J, Pluzanska A, Gorbounova V, Kaukel E, Mattson KV, Ramlau R, Szczesna A, Fidias P, Millward M, Belani CP (2003). Randomized, multinational, phase III study of docetaxel plus platinum combinations versus vinorelbine plus cisplatin for advanced non-small-cell lung cancer: the TAX 326 study group. J Clin Oncol.

[23] Shepherd FA, Dancey J, Ramlau R, Mattson K, Gralla R, O'Rourke M, Levitan N, Gressot L, Vincent M, Burkes R, Coughlin S, Kim Y, Berille J (2000). Prospective randomized trial of docetaxel versus best supportive care in patients with non-small-cell lung cancer previously treated with platinum-based chemotherapy. J Clin Oncol.

[24] Hanna N, Shepherd FA, Fossella FV, Pereira JR, De Marinis F, von Pawel J, Gatzemeier U, Tsao TC, Pless M, Muller T, Lim HL, Desch C, Szondy K (2004). Randomized phase III trial of pemetrexed versus docetaxel in patients with non-small-cell lung cancer previously treated with chemotherapy. J Clin Oncol.

[25] Adjei AA, Mandrekar SJ, Dy GK, Molina JR, Gandara DR, Ziegler KL, Stella PJ, Rowland KM, Schild SE, Zinner RG (2010). Phase II trial of pemetrexed plus bevacizumab for second-line therapy of patients with advanced non-small-cell lung cancer: NCCTG and SWOG study N0426. J Clin Oncol.

[26] Heist RS, Fidias P, Huberman M, Ardman B, Sequist LV, Temel JS, Lynch TJ (2008). A phase II study of oxaliplatin, pemetrexed, and bevacizumab in previously treated advanced non-small cell lung cancer. J Thorac Oncol.

[27] Weiss GJ, Zeng C, Kelly K, Tran ZV, Bunn PA (2007). Single-institution experience with pemetrexed and bevacizumab as salvage therapy in advanced non-small-cell lung cancer. Clin Lung Cancer.

[28] Liu KJ, Ding LY, Wu HY (2015). Bevacizumab in combination with anticancer drugs for previously treated advanced non-small cell lung cancer. Tumour Biol.

[29] Ding L, Liu K, Jiang Z, Chen Q, Zhou N, Liang Y, Gao H, Hong X, Wu H (2015). The efficacy and safety of pemetrexed plus bevacizumab in previously treated patients with advanced non-squamous non-small cell lung cancer (ns-NSCLC). Tumour Biol.

[30] Scagliotti GV, Parikh P, von Pawel J, Biesma B, Vansteenkiste J, Manegold C, Serwatowski P, Gatzemeier U, Digumarti R, Zukin M, Lee JS, Mellemgaard A, Park K (2008). Phase III study comparing cisplatin plus gemcitabine with cisplatin plus pemetrexed in chemotherapy-naive patients with advanced-stage non-small-cell lung cancer. J Clin Oncol.

[31] Nuijten MJ, Aultman R, Carpeno Jde C, Vergnenegre A, Chouaid C, Walzer S, Siebert U (2011). An indirect comparison of the efficacy of bevacizumab plus carboplatin and paclitaxel versus pemetrexed with cisplatin in patients with advanced or recurrent non-squamous adenocarcinoma non-small cell lung cancer. Curr Med Res Opin.

[32] Ogasawara T, Kasamatsu N, Umezawa H, Takeuchi T, Naito Y, Hashizume I (2011). Retrospective analysis of pemetrexed plus cisplatin chemotherapy for elderly advanced non-small-cell lung cancer. Gan To Kagaku Ryoho.

[33] Paz-Ares L, Mezger J, Ciuleanu TE, Fischer JR, von Pawel J, Provencio M, Kazarnowicz A, Losonczy G, de Castro G, Szczesna A, Crino L, Reck M, Ramlau R (2015). Necitumumab plus pemetrexed and cisplatin as first-line therapy in patients with stage IV non-squamous non-small-cell lung cancer (INSPIRE): an open-label, randomised, controlled phase 3 study. Lancet Oncol.

[34] Scagliotti GV, Park K, Patil S, Rolski J, Goksel T, Martins R, Gans SJ, Visseren-Grul C, Peterson P (2009). Survival without toxicity for cisplatin plus pemetrexed versus cisplatin plus gemcitabine in chemonaive patients with advanced non-small cell lung cancer: a risk-benefit analysis of a large phase III study. Eur J Cancer.

[35] Schmid-Bindert G, Gebbia V, Mayer F, Arriola E, Márquez-Medina D, Syrigos K, Biesma B, Leschinger MI, Frimodt-Moller B, Ripoche V, Myrand SP, Nguyen TS, Hozak RR (2013). Phase II study of pemetrexed and cisplatin plus cetuximab followed by pemetrexed and cetuximab maintenance therapy in patients with advanced nonsquamous non-small cell lung cancer. Lung cancer.

[36] Zhang GZ, Jiao SC, Meng ZT (2010). Pemetrexed plus cisplatin/carboplatin in previously treated locally advanced or metastatic non-small cell lung cancer patients. J Exp Clin Cancer Res.

[37] Barlesi F, Scherpereel A, Rittmeyer A, Pazzola A, Ferrer Tur N, Kim JH, Ahn MJ, Aerts JG, Gorbunova V, Vikström A, Wong EK, Perez-Moreno P, Mitchell L (2013). Randomized phase III trial of maintenance bevacizumab with or without pemetrexed after first-line induction with bevacizumab, cisplatin, and pemetrexed in advanced nonsquamous non-small-cell lung cancer: AVAPERL (MO22089). J Clin Oncol.

[38] Herbst RS, Johnson DH, Mininberg E, Carbone DP, Henderson T, Kim ES, Blumenschein G, Lee JJ, Liu DD, Truong MT, Hong WK, Tran H, Tsao A (2005). Phase I/II trial evaluating the anti-vascular endothelial growth factor monoclonal antibody bevacizumab in combination with the HER-1/epidermal growth factor receptor tyrosine kinase inhibitor erlotinib for patients with recurrent non-small-cell lung cancer. J Clin Oncol.

[39] Herbst RS, O'Neill VJ, Fehrenbacher L, Belani CP, Bonomi PD, Hart L, Melnyk O, Ramies D, Lin M, Sandler A (2007). Phase II study of efficacy and safety of bevacizumab in combination with chemotherapy or erlotinib compared with chemotherapy alone for treatment of recurrent or refractory non small-cell lung cancer. J Clin Oncol.

[40] Reck M, von Pawel J, Zatloukal P, Ramlau R, Gorbounova V, Hirsh V, Leighl N, Mezger J, Archer V, Moore N, Manegold C (2009). Phase III trial of cisplatin plus gemcitabine with either placebo or bevacizumab as first-line therapy for nonsquamous non-small-cell lung cancer: AVAil. J Clin Oncol.

